# Ivor Lewis minimally invasive oesophagectomy versus McKeown approach: short-term benefits and mid-term equivalence in a randomized trial for oesophageal squamous cell carcinoma

**DOI:** 10.1007/s00464-025-12424-7

**Published:** 2025-12-04

**Authors:** Ruipu Xiu, Ran An, Lei Shan, Weiquan Zhang, Bo Cong, Xiaogang Zhao, Yunpeng Zhao

**Affiliations:** 1 The Second Clinical Medical College, Cheeloo College of Medicine, Shandong University, Jinan, China; 2 Department of Thoracic Surgery, The Second Qilu Hospital of Shandong University, Jinan, China

**Keywords:** Oesophageal cancer, Minimally invasive oesophagectomy, Ivor Lewis, McKeown, Prospective randomized controlled trial

## Abstract

**Background:**

Oesophageal cancer (EC) is a leading cause of cancer-related mortality worldwide. Minimally invasive oesophagectomy (MIE) techniques—such as the Ivor Lewis and McKeown techniques—are widely used to treat mid-lower thoracic oesophageal squamous cell carcinoma (ESCC). However, the comparative efficacy of these techniques remains debated, particularly with respect to mid-term outcomes. This study aimed to compare the short- and mid-term outcomes of Ivor Lewis versus McKeown MIE in patients with mid-lower ESCC.

**Materials and Methods:**

This prospective randomized controlled trial (July 2020 – June 2024) enrolled 272 ESCC patients at a single Chinese centre. The patients were randomized to the Ivor Lewis (*n* = 136) or McKeown (*n* = 136) MIE groups at a 1:1 ratio. The primary endpoint was postoperative complications; the secondary endpoints included operative parameters, laboratory biomarkers, and progression-free survival (PFS). Statistical analyses, including Kaplan–Meier survival analysis, were performed using SPSS 29.0 and R 4.4.2.

**Results:**

The Ivor Lewis group demonstrated significantly lower complication rates and shorter operative time (median [IQR] 210 [176–240] vs. 285 [245–335] minutes, Mann–Whitney U test, *p* < 0.001), including the rates of anastomotic leak (8.1 vs. 16.9%, *p* = 0.03), anastomotic stenosis (6.6% vs. 22.8%, *p* < 0.001), and recurrent laryngeal nerve injury (0 vs. 3.7%, *p* = 0.02). Both groups exhibited comparable postoperative inflammatory (WBC and CRP) and nutritional (albumin and prealbumin) laboratory changes. No significant difference in PFS was observed (log-rank *p* = 0.67).

**Conclusion:**

Compared with the McKeown approach, Ivor Lewis MIE yields superior short-term outcomes, including reduced operative time and complications. However, there was no significant difference in mid-term survival (as measured by PFS) between the groups.

**Graphical Abstract:**

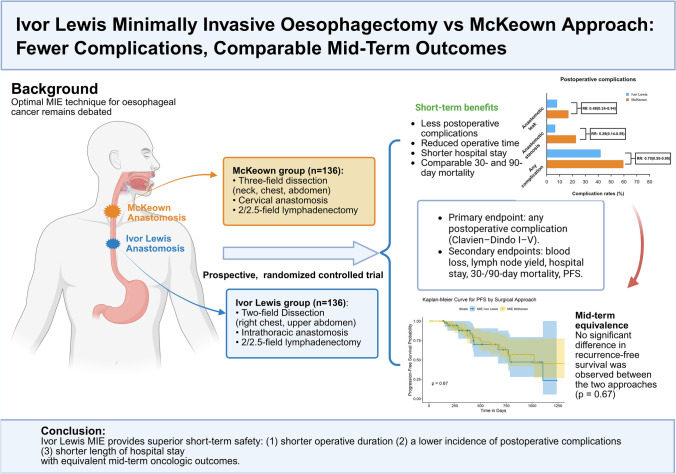

**Supplementary Information:**

The online version contains supplementary material available at 10.1007/s00464-025-12424-7.

Oesophageal cancer (EC) remains a major public health challenge worldwide, ranking seventh in incidence and sixth in mortality worldwide in 2020 [1]. Oesophageal squamous cell carcinoma (ESCC) is the predominant histological subtype, and 87% of ESCC cases involve the proximal to middle oesophagus [1]. In recent decades, the incidence of EC has been increasing in various regions, including Eastern Europe and North America, driven in part by lifestyle factors such as smoking, alcohol consumption, and poor dietary habits [2, 3]. Gastroesophageal reflux disease (GERD), a condition more prevalent in Western populations [4], is a well-established risk factor for the development of oesophageal adenocarcinoma and contributes to the increasing incidence of this malignancy in Western countries [5]. Despite continuous advancements in medical technology, the 5-year survival rate for patients with EC remains low, typically ranging from 15 to 25% in most countries, owing to late-stage diagnosis and limited treatment options [6].

In particular, China accounts for the highest absolute number of EC cases amongst all countries and is responsible for approximately half of the global deaths from this disease [7, 8]. The burden of EC in China is disproportionate, thus making this disease a critical area of concern for both public health and medical research [9]. The increasing prevalence of EC in China is driven by a combination of traditional risk factors, such as smoking and alcohol consumption, dietary habits, and environmental factors [10].

Radical surgery, including complete tumour resection and lymph node dissection after neoadjuvant chemotherapy or chemoradiotherapy, remains the standard treatment for EC [11]. Since the introduction of thoracoscopic oesophagectomy by Cuschieri in 1992 [11], minimally invasive oesophagectomy (MIE) has gradually become the procedure of choice owing to its safety and efficacy in terms of achieving radical resection [12]. In recent decades, there have been significant advances in the field, particularly with the introduction of robotic-assisted surgery. The combination of thoracoscopic and laparoscopic techniques has increased surgical precision, reduced surgical trauma, and improved short-term outcomes, such as decreased blood loss and faster recovery times [13, 14]. These advances have led to broader adoption of MIE across clinical settings, thereby contributing to improved patient outcomes.

The two most common MIE approaches, Ivor Lewis and McKeown oesophagectomies, differ primarily in the location of the anastomosis—cervical in McKeown and intrathoracic in Ivor Lewis [15]. Debate continues over which procedures provide superior mid-term outcomes in survival and quality of life whilst both are widely adopted. McKeown MIE enables radical lymphadenectomy and wider proximal margins, critical for mid-upper oesophageal tumours, at the cost of higher cervical complications (leak, stenosis) and prolonged recovery [16]. In contrast, Ivor Lewis MIE reduces complications (anastomotic leaks, recurrent laryngeal nerve [RLN] palsy) and shortens operative time [17]. Retrospective studies have suggested favourable short-term outcomes with Ivor Lewis, but the lack of mid-term survival data limits conclusions on overall prognosis [17, 18]. Moreover, it is worth paying attention to the associations between perioperative laboratory markers and surgical approaches—an important aspect for comprehensively assessing patient status during the perioperative period.

This prospective randomized controlled trial aims to address this gap by comparing the Ivor Lewis and McKeown procedures with respect to perioperative complications (e.g., anatomic leak, anatomic stenosis), laboratory findings, mid-term survival and disease progression. Additionally, our study included a comprehensive subgroup analysis, providing valuable insights into how these statuses are correlated with different surgical approaches.

## Methods

### Clinical information

This prospective, randomized controlled trial included 272 patients diagnosed with EC who were admitted to a single medical centre (The Second Qilu Hospital of Shandong University, China) between July 2020 and June 2024. The study was initiated in mid-2020 under institutional oversight and received ethics approval from the Ethics Committee of the Second Hospital of Shandong University (approval No. SDDXDEYY-KYB2019-1189), consistent with the ClinicalTrials.gov registration (NCT04217239). This study aimed to compare outcomes between two minimally invasive oesophagectomy (MIE) procedures that are used to treat mid-lower thoracic ESCC, with 136 patients in the Ivor Lewis group and 136 patients in the McKeown group.

The inclusion criteria were as follows: (1) Patients with clinically staged T_1-3_N_0-2_M0 tumours; good cardiopulmonary function; (2) Patients with lower thoracic oesophageal tumours and oesophageal-gastric junction tumour; (3) Patients without a previous history of cancer; (4) Patients without a previous history of neck or chest surgery. The exclusion criteria were as follows: (1) cardiopulmonary function not good enough for surgery; (2) Patients with hybrid MIE.

**Fig. 1:**
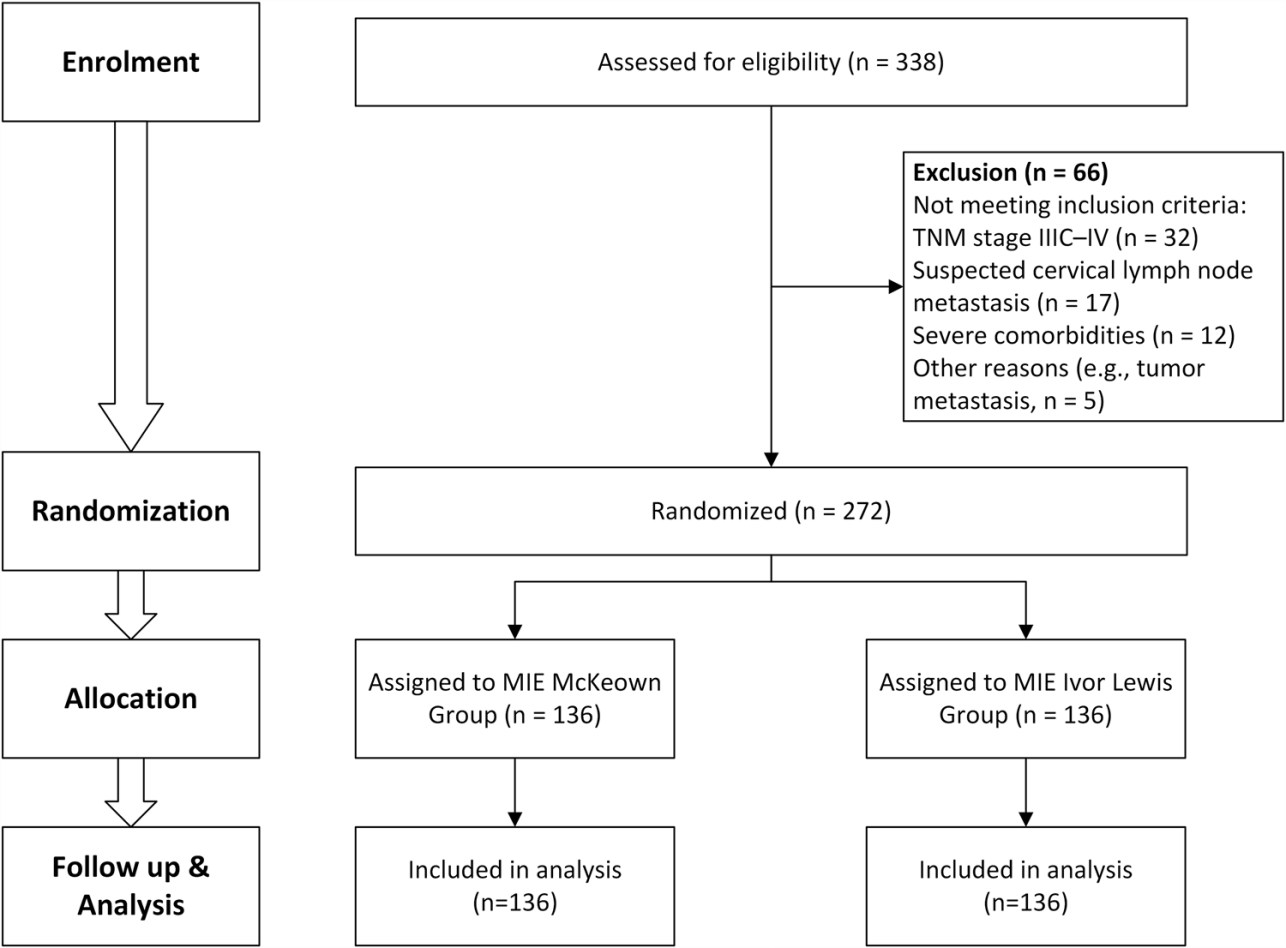
CONSORT diagram for the trial. *CONSORT* consolidated standards of reporting trials, *TNM* tumour node metastasis, *MIE* minimally invasive oesophagectomy

Figure 1 shows a CONSORT flowchart. Of the 338 consecutive patients screened for eligibility, 66 were excluded on the basis of the exclusion criteria. The remaining 272 patients were randomized to the two MIE groups using a computer-generated sequence: 136 to the Ivor Lewis group and 136 to the McKeown group. The baseline characteristics of the patients included age, sex, body mass index (BMI), smoking status, hypertension, diabetes mellitus, coronary heart disease (CHD), and cerebrovascular disease (CVD). Both groups adhered to the study protocol throughout the trial. All randomized participants were included in the final intention-to-treat analysis.

### Surgical methods

All patients underwent minimally invasive oesophagectomy under general anaesthesia with single-lumen endotracheal intubation to optimise exposure of the upper mediastinum. Two standard surgical approaches were employed: the McKeown and Ivor Lewis procedures, selected based on tumour location and clinical assessment. The McKeown procedure involved thoracoscopic mobilisation of the oesophagus, laparoscopic gastric conduit formation, and cervical anastomosis. The Ivor Lewis procedure combined laparoscopic gastric mobilisation with thoracoscopic oesophageal dissection and intrathoracic anastomosis.

**Fig. 2:**
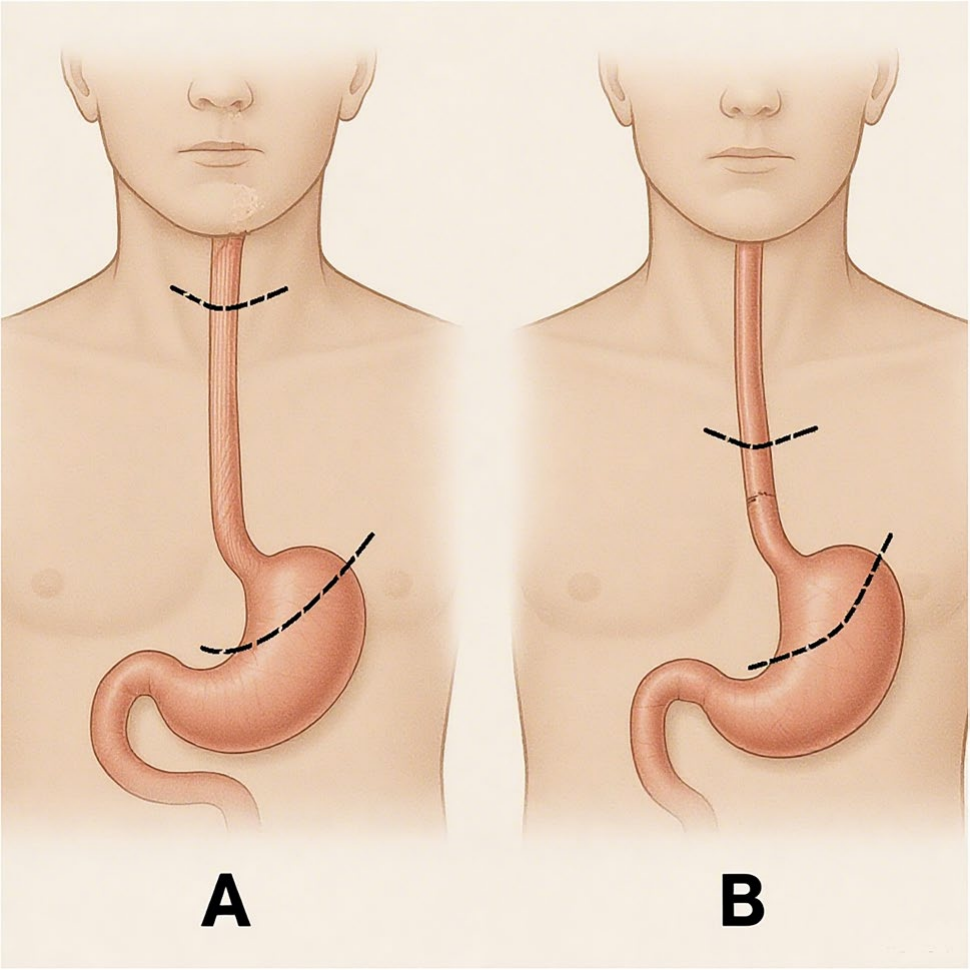
Schematic illustration of McKeown (**A**) and Ivor Lewis (**B**) oesophagectomy procedures. The McKeown approach involves resection of the oesophagus and gastric cardia via a cervical anastomosis, whilst the Ivor Lewis procedure entails intrathoracic anastomosis following resection. Skin incisions (e.g., cervical incision and mini-thoracotomy) are not depicted

Detailed surgical steps, intraoperative anatomical landmarks, lymphadenectomy protocols, and anastomotic techniques are provided in Supplementary Materials and Fig. 2.

### Endpoints

The primary endpoint of this study was the overall postoperative complications within 30 days.. The secondary endpoints included operative time, blood loss, number of lymph node dissections, three-day postoperative laboratory findings, length of hospital stay, operative-related mortality within 3 months after surgery, and progression-free survival (PFS).

Postoperative complications were defined according to the Oesophagectomy Complications Consensus Group (ECCG) and International Society for Diseases of the Oesophagus (ISDE) criteria. Severity was graded using the Clavien–Dindo classification. All complications occurring within 30 days after surgery or during the index hospitalization (whichever was longer) were included. Postoperative complications were independently reviewed by three senior thoracic surgeons who were not involved in the index operation. The reviewers were blinded to the surgical approach during event adjudication. Patients with incomplete follow-up or missing data for the analysed endpoint were excluded from the corresponding subgroup or survival analyses.

Serious adverse events (SAEs) were defined as life-threatening complications requiring intensive care, reoperation, or resulting in death (corresponding to Clavien–Dindo grade IV–V). All adverse events were recorded prospectively and reported in accordance with the CONSORT extension for reporting harms in randomized trials.

### Statistical analysis

Statistical analysis was conducted using the SPSS software (version 29.0). R (version 4.4.2) was used for initial data cleaning and for calculating and visualizing the odds ratios (ORs) for subgroup analyses. Continuous data that are not normally distributed are typically expressed as medians and interquartile ranges, and they were compared using the two-sample Wilcoxon rank-sum test. The chi-square test or Fisher's exact test was used to compare the incidence rates such as postoperative complication rates. For survival analysis, the Kaplan–Meier method (K–M method) was used to estimate survival rates, and the log-rank test (a nonparametric test) was employed to compare survival rates between groups. A two-sided *p *value < 0.05 was considered statistically significant for all analyses.

The sample size was calculated based on the primary endpoint of overall postoperative complications within 30 days, defined by the Clavien–Dindo classification. According to published literature and our preliminary institutional data, we assumed a baseline incidence of 35% in the McKeown group and 20% in the Ivor Lewis group. Using a two-sided test with α = 0.05 and power = 0.80, and applying the formula

$$n = \frac{{p_{1} \left( {1 - p_{1} } \right) + p_{2} \left( {1 - p_{2} } \right)}}{{\left( {p_{2} - p_{1} } \right)^{2} }} \times f\left( {\alpha ,\beta } \right)$$

where $f\left( {\alpha ,\beta } \right) = 7.9$, the required sample size per arm was 136 patients. No interim analyses or α spending adjustments were planned.

### Subgroup analysis

Prespecified subgroup analyses were conducted to evaluate complication rates across demographic and clinical characteristics, including age (≤ 60 vs. > 60 years), sex (male vs. female), BMI (< 25 vs. ≥ 25 kg/m^2^), smoking status (ever/never), alcohol consumption (ever/never), hypertension (yes/no), diabetes (yes/no), and receipt of neoadjuvant therapy (yes/no). Patients were stratified by age using a 60-year cut-off, aligning with the World Health Organization's definition of older adults, and by body mass index using WHO categories for overweight status. Subgroup differences were assessed using multivariable logistic regression models adjusted for baseline covariates (e.g., tumour stage), with odds ratios (ORs) and 95% confidence intervals (CIs) calculated. Interaction effects between surgical approach and subgroups were tested using likelihood ratio tests.

## Results

### Patient characteristics

A total of 272 patients were analysed, with 136 in the McKeown group and 136 in the Ivor Lewis group. Baseline characteristics, including age, sex, body mass index (BMI), smoking status, hypertension, diabetes mellitus, coronary heart disease (CHD), and cerebrovascular disease (CVD), were similar across the groups (Table 1). The median age was 65 years (interquartile range [IQR], 59–70 years) in the McKeown group and 67.5 years (IQR, 61–73 years) in the Ivor Lewis group. Sex distribution was similar (78.7% vs. 83.8% male, *p* = 0.28). BMI (22.98 vs. 23.19, *p* = 0.06), smoking status (35.3% vs. 36.8% ever, *p* = 0.14), and drinking status (39.0% vs. 44.1% ever,* p* = 0.71) did not differ significantly. Though hypertension (19.9% vs. 27.2%) and diabetes mellitus (7.4% vs. 11.8%) were more common in the Ivor Lewis group, differences were not statistically significant, (*p* = 0.15, *p* = 0.22, respectively).

**Table 1:** Comparison of baseline information between the two groups [cases (%)/median (i.q.r)]

Characteristics	McKeown MIE group (*n* = 136)	Ivor Lewis MIE group (*n* = 136)	*p* value
Age, years			0.06
Median	65	67.5	
Interquartile range	59–70	61–73	
Range	41–80	45–84	
Gender, n (%)			0.28
Male	107 (78.7)	114 (83.8)	
Female	29 (21.3)	22 (16.2)	
BMI^a^, kg/m^2^			0.06
Median	22.98	23.19	
Interquartile range	59–70	61–73	
Range	16.33–31.25	16.90–30.86	
Smoking status, n (%)			0.14
Ever	48 (35.3)	50 (36.8)	
Never	88 (64.7)	86 (63.2)	
Drinking status, *n* (%)			0.71
Ever	53 (39.0)	60 (44.1)	
Never	83 (61.0)	76 (55.9)	
Comorbidity			
Hypertension	27 (19.9)	37 (27.2)	0.15
Diabetes mellitus	10 (7.4)	16 (11.8)	0.22
CHD^b^	6 (4.4)	2 (1.5)	0.25
CVD^c^	1 (0.7)	4 (2.9)	0.18
Pathological staging			
I	28(20.6)	34(25.0)	0.75
II	53(39.0)	41(30.1)	0.07
III	55(40.4)	61(44.9)	0.62
Pulmonary function			
FEV1 (L) ^d^			0.15
Median	2.69	2.44	
Interquartile range	2.06–3.18	2.05–2.95	
Range	1.11–4.35	1.03–4.23	
MVV (L)^e^			0.50
Median	88.9	83.0	
Interquartile range	69.5–114.9	71.0–104.0	
Range	31.8–153.6	34.9–142.7	
Cardiovascular function			
LVEF (%)^f^			0.49
Median	64	63	
Interquartile range	61–66	60–66	
Range	42–74	54–74	
Neoadjuvant therapy			0.32
No	100(73.5)	107(78.7)	
Yes			
Chemotherapy	7(5.2)	8(5.9)	
Chemoradiotherapy	29(21.3)	21(15.4)	
Neoadjuvant therapy cycles			0.13
Median	2	2	
Interquartile range	2–2	2–2	
Range	1–5	1–2	
Adjuvant therapy			0.005
No	59(43.4)	82(60.3)	
Yes			
Chemotherapy	51(37.5)	44(32.4)	
Chemoradiotherapy	26(19.1)	10(7.4)	
Neoadjuvant therapy cycles			< 0.001
Median	2	4	
Interquartile range	1–5	2.25–5	
Range	1–7	1–9	

^a^*BMI* body mass index, ^b^*CHD* coronary heart disease, ^c^*CVD* cerebrovascular disease, ^d^*FEV1* forced expiratory volume in 1.0 s, ^e^*MVV* maximum volume for ventilation, ^f^*LVEF* left ventricular ejection fraction

### Surgical outcomes

The surgical outcomes between the McKeown and Ivor Lewis groups were compared, and notable differences were observed. The median operation time was significantly longer in the McKeown group than in the Ivor Lewis group (285 min, IQR, 245–335 min vs. 210 min, IQR, 176–240 min; *p* < 0.001). This substantial difference in operation time suggested that the McKeown procedure is more time-consuming. There were no significant differences in intraoperative blood loss between the two groups (*p* = 0.32). Similarly, the number of dissected lymph nodes, an important marker of the extent of surgery, did not differ significantly between the two groups (*p* = 0.14).

Postoperative complications were also analysed, and several significant differences were observed between the groups. The McKeown group had significantly higher incidence rates of anastomotic leak (16.9% vs. 8.1%, *p* = 0.03) and stenosis (22.8% vs. 6.6%, *p* < 0.001). Additionally, RLN palsy occurred more frequently in the McKeown group (*p* = 0.02). Other complications, such as surgical site infection (SSI) and unplanned reoperation, were similar between the two groups (*p* = 0.05 and *p* = 0.47, respectively). The incidence of pulmonary infection was not significantly different between the two groups (*p* = 0.58). The length of stay (LOS) was significantly shorter for patients in the Ivor Lewis group than those in the McKeown group (16 days, IQR, 14–22 days vs. 19 days, IQR, 15–31 days; *p* < 0.001). Mortality rates within 180 days postsurgery did not differ significantly between the groups (*p* = 0.49).

### Blood laboratory examination results

Both groups exhibited significant postoperative changes in laboratory parameters, reflecting surgical stress and nutritional impact. In the McKeown group, serum albumin levels declined from a median of 41.10 g/L preoperatively to 36.60 g/L postoperatively (*p* < 0.001). A similar reduction was observed in the Ivor Lewis group, from 41.55 g/L to 36.70 g/L (*p* < 0.001). However, the extent of decline was comparable between the two groups (*p* = 0.947). Prealbumin and globulin levels also decreased significantly after surgery in both groups, with no statistically significant difference in the degree of change between them (PA: *p* = 0.866; Globulin: *p* = 0.855).

Liver enzyme levels increased markedly postoperatively. In the McKeown group, ALT rose from 13.00 to 20.00 U/L and AST from 17.00 to 32.00 U/L (both *p* < 0.001). The Ivor Lewis group showed similar increases (ALT: 11.20 to 18.00 U/L; AST: 16.00 to 34.50 U/L; both *p* < 0.001). The changes in ALT and AST levels were not significantly different between the groups (ALT: *p* = 0.127; AST: *p* = 0.293).

Postoperative inflammatory responses were evident in both groups. White blood cell counts rose significantly (McKeown: 5.73 to 12.53 × 10⁹/L; Ivor Lewis: 5.55 to 11.86 × 10⁹/L; both *p* < 0.001), as did neutrophil counts (McKeown: 3.52 to 10.68 × 10⁹/L; Ivor Lewis: 3.44 to 10.30 × 10⁹/L; both *p* < 0.001). Again, the magnitude of increase was similar between groups (WBC: *p* = 0.151; NEUT: *p* = 0.095). C-reactive protein (CRP) levels increased postoperatively in both groups, with a slightly higher median in the McKeown group (83.35 vs. 75.35 mg/L), though the difference was not statistically significant (*p* = 0.181).

### Progression-free survival (PFS)

**Fig. 3:**
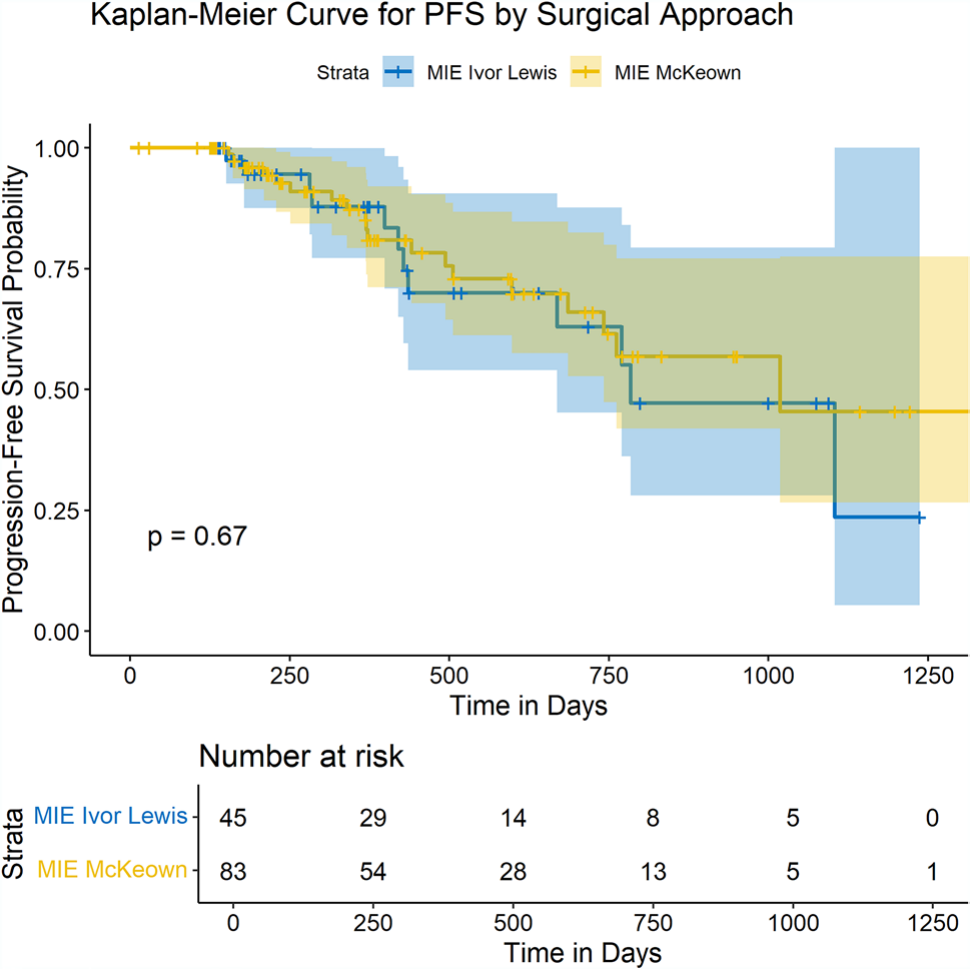
Kaplan‒Meier curve for progression-free survival (PFS) according to surgical approach. This plot compares the progression-free survival rates between patients who underwent Ivor Lewis MIE (blue) and McKeown MIE (yellow). The Kaplan‒Meier survival curves revealed no significant difference in PFS between the two surgical approaches (*p* = 0.67). The table below the curve shows the number of patients at risk at various time points. Only patients with complete follow-up data were included in this analysis (McKeown, *n* = 45; Ivor Lewis, *n* = 83)

No significant difference in PFS was observed between the Ivor Lewis group and the McKeown group (*p* = 0.67) (Fig. 3). This suggests that despite the differences in surgical outcomes and postoperative complications, both approaches resulted in similar mid-term disease control in terms of PFS.

### Subgroup analysis

**Fig. 4:**
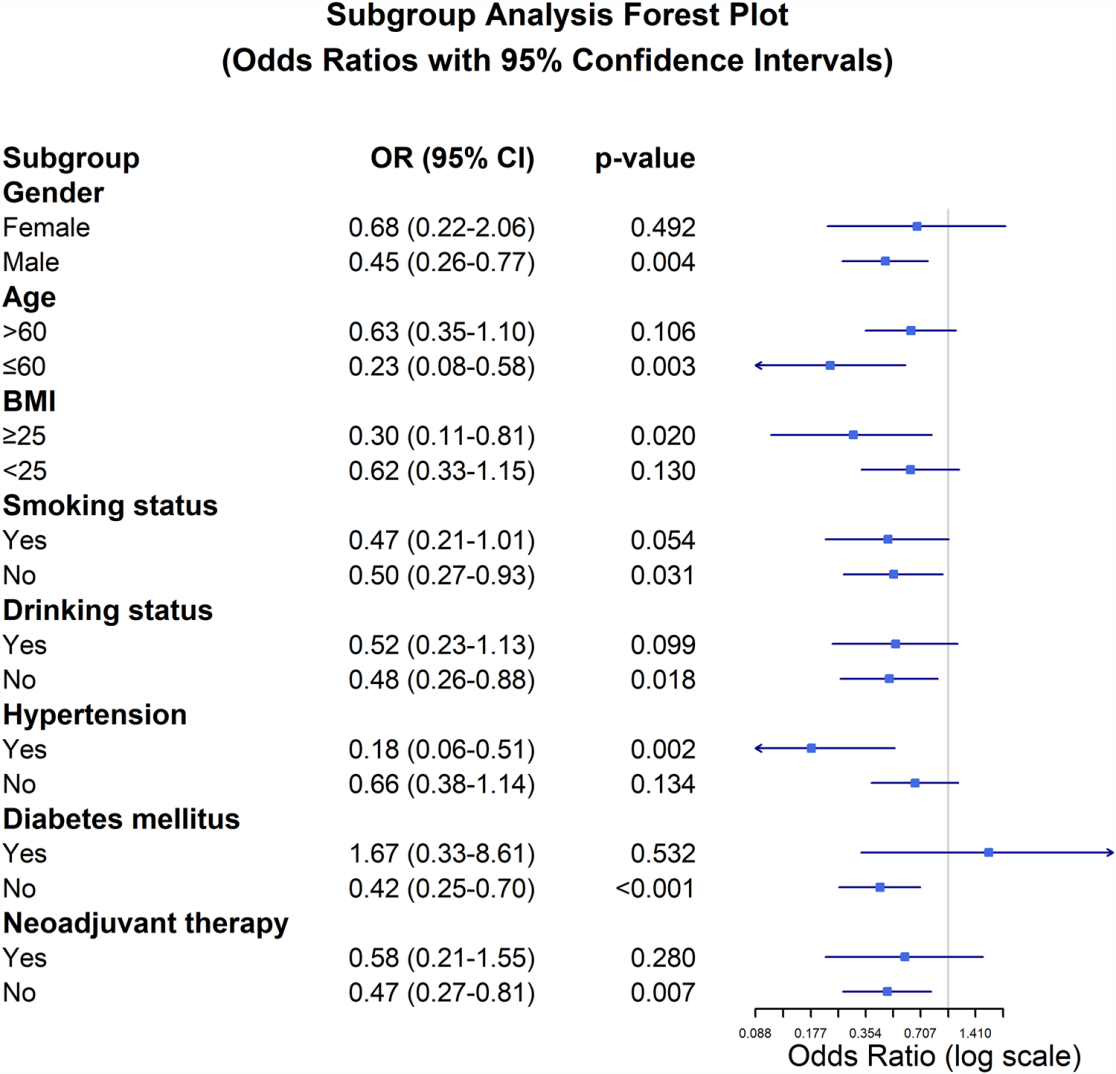
Subgroup analysis forest plot of odds ratios (ORs) with 95% confidence intervals (CIs) for complications following McKeown MIE and Ivor Lewis MIE. An OR less than 1 indicates a better outcome with Ivor Lewis MIE. Significant p values are indicated for each subgroup

Subgroup analysis revealed that the Ivor Lewis procedure was associated with a lower risk of postoperative complications in several patient subpopulations (Fig. 4). Fewer complications were observed in males (OR 0.45, 95% CI: 0.26–0.77; *p* = 0.004), whereas no significant reduction was observed amongst females (OR 0.68, 95% CI: 0.22–2.06; *p* = 0.492). Similarly, patients aged ≤ 60 years showed fewer complications with Ivor Lewis (OR 0.23, 95% CI: 0.08–0.58; *p* = 0.003), whilst those > 60 years had no significant benefit (OR 0.63, 95% CI: 0.35–1.10; *p* = 0.106) compared with that in the McKeown approach. Patients with BMI ≥ 25 kg/m^2^ had lower complication rate following the Ivor Lewis procedure (OR 0.30, 95% CI: 0.11–0.81; *p* = 0.020), whereas those with BMI < 25 kg/m^2^ experienced no significant difference between the two approaches (OR 0.62, 95% CI: 0.33–1.15; *p* = 0.130).

Nonsmokers had fewer complications (OR 0.50, 95% CI: 0.27–0.93; *p* = 0.031) with the Ivor Lewis approach, whilst smokers showed a borderline trend favouring Ivor Lewis (OR 0.47, 95% CI: 0.21–1.01; *p* = 0.054). Similarly, nondrinkers derived a significant benefit (OR 0.48, 95% CI: 0.26–0.88; *p* = 0.018) from the Ivor Lewis procedure, whereas drinkers showed no significant intergroup difference (OR 0.52, 95% CI: 0.23–1.13; *p* = 0.099).

Amongst hypertensive patients, the Ivor Lewis procedure reduced postoperative complications (OR 0.18, 95% CI: 0.06–0.51; *p* = 0.002), whilst no significant effect was observed in normotensive individuals (OR 0.66, 95% CI: 0.38–1.14; *p* = 0.134). Non-diabetic patients improved significantly (OR 0.42, 95% CI: 0.25–0.70; *p* < 0.001), but not in those with diabetes (OR 1.67, 95% CI: 0.33–8.61;* p* = 0.532).

Regarding neoadjuvant therapy, patients who did not receive neoadjuvant treatment experienced a significantly lower rate of postoperative complications following the Ivor Lewis procedure (OR 0.47, 95% CI: 0.27–0.81; *p* = 0.007), whereas amongst those who received neoadjuvant therapy, the difference between the two approaches was not statistically significant (OR 0.58, 95% CI: 0.21–1.55; *p* = 0.280).

## Discussion

This prospective, randomized controlled trial provided a comprehensive comparison of the perioperative outcomes and survival profiles of McKeown and Ivor Lewis MIE. The endpoints included operative time, intraoperative blood loss, number of lymph nodes dissected, postoperative complications, laboratory parameters, and progression-free survival (PFS). Subgroup analysis was used to evaluate heterogeneity in treatment efficacy across demographic subpopulations. The Ivor Lewis procedure was associated with favourable short-term outcomes, notably shorter operative duration and a lower incidence of postoperative complications. However, no significant difference in PFS was observed between the two techniques. These findings highlight the importance of tailoring surgical strategy to individual patient characteristics and anatomical context.

With respect to intraoperative parameters, the Ivor Lewis procedure was associated with a significantly shorter operative time compared to the McKeown approach. This difference likely reflects the streamlined nature of the two-field dissection in Ivor Lewis, as opposed to the more extensive three-field configuration required in McKeown. Enhanced procedural efficiency may also be attributed to more straightforward conduit preparation and intrathoracic anastomosis. However, variability in operative time has been reported across studies, highlighting the influence of surgeon experience and institutional protocols [19, 20]. Despite these differences, both procedures maintained comparable intraoperative blood loss and total lymph node dissection. Importantly, the frequent pairing of the McKeown procedure with three-field dissection reflects the procedural advantage of cervical access [21]. However, emerging evidence suggests that oncological outcomes may be equivalent between two- and three-field strategies, reinforcing the importance of individualized surgical planning based on patient-specific factors, including appropriate perioperative management and adjunctive therapies [22].

Postoesophagectomy complications primarily include anastomotic leak, stricture, and RLN palsy. Pulmonary infection has emerged as a more serious threat than anastomotic leak, with reported incidences ranging from 10 to 30% [23]. In our study, the overall complication burden was significantly higher in the McKeown cohort, particularly with respect to fistula formation (*p* = 0.028), which may be attributable to anatomical and technical factors. Cervical anastomosis in McKeown necessitates greater gastric conduit mobilization, resulting in increased biomechanical tension and impairing microvascular perfusion—both critical contributors to anastomotic breakdown. Moreover, extensive lymph node dissection often reduces soft tissue coverage at the anastomotic site, further elevating the risk of leakage. In contrast, the Ivor Lewis procedure, which employs an intrathoracic anastomosis, reduces conduit elevation, preserves vascular supply, and minimizes anastomotic tension [17]. Although thoracic fistulas occurred less frequently, their mediastinal location is associated with higher morbidity due to complications such as pyothorax, haemorrhage, sepsis, and multiorgan failure. Conversely, cervical fistulas, usually result in localized cervical abscess due to anatomical containment [24]. Fistulas are also key risk factors for anastomotic stricture [25, 26]. A previous meta-analysis further identified cardiovascular disease and diabetes as additional contributors to stricture formation [27]. Although the molecular mechanisms remain unclear, it is hypothesized that fibrosis secondary to fistula-associated inflammation and subsequent scar formation plays a central role [28]. Consistent with this, our findings demonstrated a higher incidence of anastomotic stricture in the McKeown group, paralleling the increased fistula rates. RLN palsy was also more common following McKeown. Notably, the actual extent of dissection in both groups, especially the Ivor Lewis group, was generally limited to two or two-and-a-half fields, depending on tumour level and the surgeon’s discretion. The McKeown approach requires a left cervical incision for the oesophagogastric anastomosis, which necessitates additional dissection in the narrow anatomical space adjacent to the left recurrent laryngeal nerve (RLN). This close relationship increases the likelihood of traction or thermal injury during cervical manipulation. By contrast, the Ivor Lewis procedure performs an intrathoracic anastomosis, where the anastomotic level varies with tumour height, and surgeons often adopt a more conservative dissection strategy in the upper mediastinum to avoid excessive RLN exposure. These anatomical and procedural distinctions may therefore explain the higher incidence of RLN palsy observed with McKeown compared with Ivor Lewis. Notably, pulmonary infection rates did not differ significantly between the two groups, potentially due to a range of confounding factors such as baseline nutritional status, comorbidities (e.g., diabetes, chronic obstructive pulmonary disease, pneumoconiosis) and postoperative management strategies[29–32].

Postoperative blood tests revealed significant changes in several biomarkers in both the McKeown and Ivor Lewis groups. Notably, serum ALB, PA, and Glob levels declined following surgery, reflecting the impact of surgical stress on nutritional status. However, no statistically significant differences were observed between the two groups in this regard. Additionally, WBC and NEUT counts increased markedly in both groups (*p* < 0.001), indicating a pronounced postoperative inflammatory response. Elevated levels of C-reactive protein (CRP) were also detected; nevertheless, the extent of CRP elevation did not differ significantly between the two procedures. Collectively, these findings suggest that both surgical techniques induce comparable physiological responses in terms of inflammation, nutritional decline, and hepatic function.

Both procedures demonstrated comparable PFS rates, aligning with findings from previous multicentre studies [33, 34]. This reinforces the notion that mid-term oncological outcomes are predominantly influenced by tumour biology and the integration of multimodal therapies, rather than the choice of surgical technique, despite marked differences in perioperative outcomes. Notably, emerging evidence associates the McKeown procedure with compromised swallowing-related quality-of-life indices [35], whilst the optimal extent of lymphadenectomy remains a subject of ongoing debate [36–38]. The two-field *versus* three-field dissection strategy explored in this study merits further validation in larger cohorts.

Subgroup analysis indicated that the benefits of Ivor Lewis MIE varied across patient characteristics. Patients aged ≤ 60 years and with BMI ≥ 25 kg/m^2^ experienced notably fewer complications with the Ivor Lewis approach, suggesting that younger individuals, with better physiological reserves, may tolerate thoracic anastomosis and associated stress more effectively [39–41]. In contrast, for patients > 60 years and BMI < 25 kg/m^2^, no significant difference in complication rates was observed, indicating that advanced age may attenuate the potential benefits of Ivor Lewis. Nonsmokers and nondrinkers had significantly fewer complications with the Ivor Lewis procedure, potentially due to better tissue healing capacity and fewer comorbid insults. In contrast, amongst smokers and drinkers, the advantage of Ivor Lewis was attenuated or non-significant, possibly reflecting persistent baseline inflammatory or microvascular dysfunction [42]. Interestingly, hypertensive patients showed marked benefit with Ivor Lewis, suggesting that intrathoracic anastomosis may impose less cardiovascular strain than the more extensive dissection required for cervical anastomosis. Amongst normotensive patients, however, the choice of surgical approach appeared less critical. Diabetes mellitus emerged as a potential effect modifier. Non-diabetic patients experienced significantly fewer complications with Ivor Lewis, whereas diabetic patients derived no clear benefit—likely due to compromised microvascular function and impaired wound healing, which negate the technical advantages of the procedure. These findings highlight the importance of patient selection in surgical planning. Ivor Lewis MIE appears particularly suitable for younger, male, overweight, hypertensive, and non-smoking/nondrinking patients without diabetes. In contrast, for patients without these favourable characteristics, the benefits of Ivor Lewis over McKeown may be less pronounced, and surgical decisions should be individualized. Moreover, subgroup analysis indicated that neoadjuvant therapy did not significantly modify the comparative safety profiles of the two surgical approaches. Amongst patients who did not receive neoadjuvant therapy, the Ivor Lewis procedure was associated with a significantly lower complication rate, whereas this advantage was not observed in those who underwent preoperative chemotherapy or chemoradiotherapy. These findings suggest that the reduced morbidity with Ivor Lewis MIE is more evident in patients without preoperative treatment, whereas neoadjuvant therapy may attenuate these differences by altering tissue integrity and postoperative recovery dynamics Table 2.

**Table 2:** Surgical data of patients [cases (%)/median (i.q.r)]

Characteristics	McKeown MIE group (*n* = 136)	Ivor Lewis MIE group (*n* = 136)	*p* value
Operation time (minutes)			< 0.001
Median	285	210	
Interquartile range	245–335	176–240	
Range	180–540	135–457	
Intraoperative blood loss (mL)			0.32
Median	100	100	
Interquartile range	85–120	95–145	
Range	20–500	50–400	
Dissected lymph nodes			0.14
Median	21	20	
Interquartile range	15–30	14–27	
Range	5–71	7–56	
#106 recR	105	101	0.57
#106 recL	76	70	0.54
#107	113	104	0.17
#108, #109	72	79	0.46
#1, #3	63	67	0.72
#7	46	52	0.45
Postoperative complications			
Any complication	81(59.6)	57(41.9)	0.004
Anastomotic leak	23(16.9)	11(8.1)	0.03
Grade I	2(8.7)	1(9.0)	–
Grade II	13(56.5)	5 (45.5)	–
Grade III	8(34.8)	5 (45.5)	–
Anastomotic stenosis	31(22.8)	9(6.6)	< 0.001
RLN palsy	5(3.7)	0	0.02
Grade I	1 (20)	0	–
Grade II	2 (40)	0	–
Grade III	1 (20)	0	–
Grade IV	1 (20)	0	–
Surgical site infection	8(5.9)	2(1.5)	0.05
Unplanned reoperation^a^	5(3.7)	3(2.2)	0.47
Pulmonary infection	28(20.6)	27(19.9)	0.58
Blood transfusion	20 (14.7)	26 (19.1)	0.33
ICU	16(11.8)	23(16.9)	0.23
Length of stay (days)			< 0.001
Median	19	16	
Interquartile range	15–31	14–22	
Range	7–63	9–75	
30-day mortality	3(2.2)	1(0.7)	0.31
90-day mortality	4(2.9)	1(0.7)	0.49

a. Unplanned reoperation refers to any unanticipated return to the operating room due to postoperative complications during the index hospitalization. Complications were defined according to ECCG/ISDE consensus criteria and graded by Clavien–Dindo classification (Supplementary Table S1, S2)

The Ivor Lewis procedure demonstrated superior short-term perioperative outcomes compared to the McKeown approach, including reduced postoperative morbidity and faster recovery. However, mid-term oncological efficacy was comparable between the two techniques. Whilst these findings highlight the short-term advantages of Ivor Lewis, its technical complexity—particularly during laparoscopic conduit construction and intrathoracic anastomosis—demands advanced surgical expertise, thereby increasing operative risk and resource utilization [43]. These technical demands may temper surgical enthusiasm for its widespread adoption, contributing to the continued predominance of McKeown. Surgical decision-making should therefore be guided by a combination of factors, including patient comorbidities, tumour location, and institutional expertise, prioritizing anatomical suitability over procedural conventions.

Our findings align with previous retrospective studies demonstrating that Ivor Lewis is associated with superior short-term outcomes, including lower postoperative morbidity, reduced intraoperative haemorrhage, and faster recovery [20, 43, 44]. These advantages make it particularly suitable for high-risk patients requiring optimised perioperative recovery. Although McKeown remains the preferred option in anatomically challenging cases, the equivalent mid-term survival between techniques challenges traditional assumptions of its oncological superiority. This underscores the necessity for further prospective studies with extended follow-up to evaluate long-term outcomes. Our randomised trial not only conforms to Ivor Lewis’s perioperative benefits but also contributes novel comparative survival data and subgroup analyses, thereby supporting more nuanced, patient-centred surgical decision-making.

Several limitations in this study should be acknowledged. Firstly, the median follow-up duration of 24 months restricts the assessment of overall survival and late recurrence. However, this timeframe is adequate for evaluating perioperative complications and progression-free survival (PFS), as most recurrences in ESCC occur within two years following resection. Secondly, the single-centre nature of the study may affect generalizability. Even so, the application of standardised protocols and high surgical volume (≥ 50 MIEs per year) reinforces internal validity, and our patient cohort reflects epidemiological trends seen in high-incidence regions. Thirdly, incomplete preoperative nutritional profiling limited our ability to explore the influence of baseline nutrition on surgical outcomes. Nevertheless, comparable postoperative declines in serum albumin and prealbumin between groups (Table 3) suggest that the observed outcome differences are unlikely to have been confounded by nutritional status. Future investigations should aim to involve multicentre collaborations with prolonged follow-up periods to validate these findings across more diverse populations. Mechanistic investigations are also warranted to clarify how variations in lymphadenectomy techniques interact with perioperative therapy in shaping oncologic outcomes and patient-reported quality-of-life measures.

**Table 3:** Blood laboratory examination results

Characteristics	McKeown MIE group (*n* = 136)				Ivor Lewis MIE group (*n* = 136)				*p* value
	Preoperative	Postoperative	Δ(post–pre)	p value	Preoperative	Postoperative	Δ(post–pre)	p value	
ALB^a^ (g/L)									0.95
Median	41.10	36.60	−5.00	< 0.001	41.55	36.70	−5.10	< 0.001	
Interquartile range	38.70–43.95	34.15–38.85	−8.70–0.05		39.23–43.55	34.23–39.30	−7.85–0.75		
Range	25.2–50.6	27.60–46.30	−21.10–13.20		31.60–48.40	28.90–44.70	−17.00–10.80		
PA^b^ (mg/dL)									0.87
Median	21.60	14.00	−7.10	< 0.001	21.85	14.85	−7.15	< 0.001	
Interquartile range	18.05–24.15	12.35–16.55	−10.90–2.40		19.05–25.25	12.70–17.70	−10.80–2.75		
Range	5.30–30.90	3.70–27.40	−19.70–11.40		8.00–39.20	6.30–25.90	−24.00–6.50		
Glob^c^ (g/L)									0.86
Median	24.80	22.50	−2.70	< 0.001	24.35	21.90	−2.80	< 0.001	
Interquartile range	21.50–27.70	19.80–24.50	−6.00–1.30		21.75–27.20	19.53–24.48	−5.98–2.55		
Range	15.60–37.30	15.80–37.10	−20.80–17.10		15.50–34.70	14.00–34.00	−15.40–9.10		
TBIL^d^ (mg/dL)									0.10
Median	10.40	11.50	−0.60	0.97	10.40	11.45	1.45	0.015	
Interquartile range	7.65–15.10	7.80–15.05	−5.65–5.00		7.95–12.48	8.25–17.00	−3.28–6.50		
Range	2.60–47.90	3.20–29.50	−36.40–19.40		3.30–26.60	4.20–45.20	−17.9–37.7		
ALT^e^ (U/L)									0.12
Median	13.00	20.00	9.00	< 0.001	11.20	18.00	6.00	< 0.001	
Interquartile range	9.00–16.00	16.00–38.50	−0.50–28.00		9.00–16.00	13.25–27.00	2.00–14.75		
Range	2.00–76.00	4.00–322.00	−40.00–302.00		3.00–51.00	7.00–159.00	−43.00–126.00		
AST^f^ (U/L)									0.29
Median	17.00	32.00	17.00	< 0.001	16.00	34.50	19.00	< 0.001	
Interquartile range	14.00–22.00	25.00–52.00	6.00–33.50		14.00–19.00	28.00–45.00	12.25–29.75		
Range	10.00–66.00	11.00–223.00	−37.00–199.00		9.00–54.00	12.00–194.00	−5.00–164.00		
WBC^g^ (10^9^)									0.15
Median	5.73	12.53	6.26	< 0.001	5.55	11.86	6.09	< 0.001	
Interquartile range	4.68–6.71	9.58–15.06	4.09–9.15		4.68–7.05	9.86–13.71	2.87–8.30		
Range	3.22–11.19	2.87–39.55	−0.80–34.92		2.78–18.86	2.13–26.03	−5.90–20.50		
NEUT^h^ (10^9^)									0.10
Median	3.52	10.68	7.01	< 0.001	3.44	10.30	6.43	< 0.001	
Interquartile range	2.77–4.32	8.41–13.00	4.87–9.82		2.74–4.56	8.25–12.01	4.10–8.59		
Range	1.16–8.37	2.33–36.24	−0.10–33.12		1.33–15.38	1.54–25.20	−4.15–22.40		
CRP^i^ (mg/L)									0.18
Median		83.35				75.35			
Interquartile range		67.33–189.53				48.70–128.00			
Range		0.20–277.30				1.27–221.00			

^a^*ALB* albumin, ^b^*PA* prealbumin ^c^*Glob* globulin, ^d^*TBIL* total bilirubin, ^e^*ALT* alanine transaminase, ^f^*AST* aspartate transaminase, ^g^*WBC* white blood cell, ^h^*NEUT* neutrophil granulocytes, ^i^*CRP* C-reactive protein

## Conclusions

This study provides evidence that, compared with the McKeown approach, Ivor Lewis MIE offers superior short-term outcomes, including shorter operative duration and a lower incidence of postoperative complications. Despite these advantages, mid-term PFS was comparable between the two techniques, underscoring the dominant role of tumour biology and multimodal therapy on prognosis. Future multicentre studies with extended follow-up durations are warranted to validate the mid-term survival outcomes and to optimise lymphadenectomy strategies.

## Supplementary Information

Below is the link to the electronic supplementary material.

Supplementary file1 (DOCX 56 KB)
