# Supplementary material for: Ivor Lewis minimally invasive oesophagectomy versus McKeown approach: short-term benefits and mid-term equivalence in a randomized trial for oesophageal squamous cell carcinoma
Source: Surg Endosc. 2025 Dec 4;40(3):1901–12. doi: 10.1007/s00464-025-12424-7 (PMC12971774; doi:10.1007/s00464-025-12424-7)
Supplement: Supplementary file 1 — Supplementary file1 (DOCX 56 KB) [file 464_2025_12424_MOESM1_ESM.docx]

**Supplementary materials**

**Supplement File 1. Surgical methods**

*McKeown Minimally Invasive Oesophagectomy (MIE)*

The McKeown approach was carried out in three sequential phases: thoracic, abdominal, and cervical. For the thoracic phase, patients were positioned in the left semi-prone position. Four thoracoscopic ports were introduced along the right mid-axillary to posterior-axillary line, and an artificial pneumothorax was established using carbon dioxide insufflation at 8 cm H₂O. Following entry into the thoracic cavity, the azygos vein arch was ligated and divided. The oesophagus was mobilised along its posterior aspect adjacent to the spine with anterior traction to facilitate dissection. The thoracic duct was carefully identified and preserved where feasible. The mediastinal pleura was incised beneath the azygos arch, and the oesophagus was dissected from surrounding structures including the membranous trachea, carina, left main bronchus, and pericardium. The dissection extended inferiorly to the diaphragmatic plane and superiorly to the thoracic inlet.

During the abdominal phase, patients were placed in the supine position and pneumoperitoneum was created with carbon dioxide at 12 cm H₂O. The gastrohepatic ligament was divided to expose the left gastric vessels along the superior border of the pancreas and the common hepatic artery. The left gastric artery and vein were ligated and divided. Further mobilisation of the posterior gastric space and division of the greater curvature attachments was performed. The abdominal oesophagus and the tissue surrounding the oesophagogastric junction were dissected up to the diaphragmatic hiatus.

In the cervical phase, a left-sided cervical incision was made anterior to the sternocleidomastoid muscle. After division of the platysma, partial dissection of the sternohyoid, omohyoid, and sternothyroid muscles was undertaken to expose the cervical oesophagus. The oesophagus was divided anterior to the vertebral column, and the proximal stump was ligated and disinfected. A traction suture was used to draw the oesophagus and stomach into the abdomen through the hiatus. A tubular stomach was constructed extracorporeally using a linear stapler, with reinforcement of the staple line. The gastric conduit was then delivered via the posterior mediastinum to the neck. Cervical anastomosis was performed using either hand-sewn or stapled techniques. Nasogastric and jejunal feeding tubes were inserted, haemostasis was ensured, a drainage strip was placed at the anastomotic site, and all incisions were closed in layers.

*Ivor Lewis Minimally Invasive Oesophagectomy (MIE)*

The Ivor Lewis procedure consisted of abdominal and thoracic phases. In the abdominal phase, with the patient in the supine position, pneumoperitoneum was established at a pressure of 12 cm H₂O. The gastrohepatic ligament was divided to expose the left gastric artery along the border of the pancreas and the common hepatic artery. The left gastric vessels were ligated and divided. An ultrasonic scalpel was used to dissect the posterior gastric space and separate attachments along the greater curvature. The abdominal oesophagus and cardia were mobilised up to the hiatus. A gastric conduit was then fashioned laparoscopically and left in situ. After ensuring adequate haemostasis, the incisions were closed.

In the thoracic phase, patients were placed in the left semi-prone position, and four thoracoscopic ports were inserted into the right thoracic cavity. Carbon dioxide insufflation at 8 cm H₂O was used to establish artificial pneumothorax. The azygos vein arch was ligated and divided. The oesophagus was mobilised along its posterior plane beside the vertebral column, with careful identification and preservation of the thoracic duct. The mediastinal pleura was incised beneath the azygos vein to separate the oesophagus or tumour from adjacent structures including the trachea, carina, left bronchus, and pericardium. Dissection proceeded caudally to the diaphragm. The previously prepared gastric conduit was brought into the thoracic cavity, and oesophagogastric anastomosis was completed within the chest using either an Overlap technique, a circular stapler, or hand-sewn sutures. After extensive lavage and haemostasis, mediastinal and pleural drains were placed and the thoracic incisions were closed.

*Lymphadenectomy*

Both approaches included thorough lymphadenectomy, which was performed in accordance with the JGCA guidelines[1]: thoracic stations (#105, #106recR/L, #106TBL, #107 subcarinal, #108 paraesophageal, #109, #110, #111, #112aoA) and abdominal stations (#1/2 left and right paracardial, #3/4 lesser and greater curvature, #7 left gastric artery, #8 common hepatic artery. Selective dissection was conducted at #9 celiac axis and #11 proximal splenic artery).

1. Japanese Gastric Cancer A., *Japanese Gastric Cancer Treatment Guidelines 2021 (6th edition).* Gastric Cancer, 2023. **26**(1): p. 1-25.

**Supplementary File 2. Statistical Analysis Plan for the randomized controlled trial comparing minimally invasive McKeown versus Ivor Lewis oesophagectomy**

**Study Title:** Minimally invasive McKeown versus Ivor Lewis oesophagectomy for oesophageal cancer: a randomized controlled trial
**SAP Version Number:** Version 1.0
**SAP Date:** Dec 28, 2019
**Study Registration:** ClinicalTrials.gov Identifier: NCT04217239
**Ethics Approval:** SDDXDEYY-KYB2019-1189

**Section 1: Administrative Information**

- **Trial Principal Investigator:** Prof. Yunpeng Zhao, Second Hospital of Shandong University
- **Trial Statistician:** Yunpeng Zhao, Department of Thoracic Surgery, The Second Qilu Hospital of Shandong University, Jinan, 250033, China
- **SAP Author(s):** Xiu Ruipu, The Second Clinical Medical College, Cheeloo College of Medicine, Shandong University, Jinan, China

**Revision Control:** Not applicable for Version 1.0.

**Section 2: Introduction**

**Background and Rationale**

Oesophagectomy remains a cornerstone treatment for oesophageal cancer. Minimally invasive McKeown (three-incision) and Ivor Lewis (two-incision) oesophagectomies are widely performed, but debate remains regarding their relative short-term morbidity and mid-term outcomes. This RCT aims to provide evidence from a head-to-head comparison.

**Objectives**

- **Primary objective:** To compare 30-day overall postoperative complication rates between McKeown MIE and Ivor Lewis MIE.
- **Secondary objectives:** To evaluate operative time, blood loss, number of lymph node dissections, three-day postoperative laboratory findings, length of hospital stay, operative-related 30-day and 90-day mortality after surgery, and progression-free survival (PFS).

**Section 3: Study Methods**

**3.1 Trial Design**

- **Type:** A prospective, parallel-group, randomized controlled trial
- **Allocation ratio:** 1:1 (McKeown vs Ivor Lewis)
- **Blinding:** Outcome assessors and statisticians blinded; surgeons unblinded
- **Follow-up:** 30- and 90-day perioperative outcomes, with extended oncologic follow-up

**3.2 Randomization**

Block randomization with concealed allocation. No stratification factors applicable.

**3.3 Sample Size**

Based on primary endpoint (overall postoperative complications).

- McKeown: 35%
- Ivor Lewis: 20%
- Effect size: 15% absolute risk reduction
- α = 0.05 (two-sided), power = 80%
- Formula:

$$n=\frac{p_{1}\left( 1-p_{1} \right)+p_{2}\left( 1-p_{2} \right)}{\left( p_{2}-p_{1} \right)^{2}}\times f(\alpha,\beta)$$

- Required: 136 per group, total N=272

**3.4 Interim Analyses**

No interim analyses planned; no α-spending.

**3.5 Timing of Final Analysis**

Final analysis will occur once all patients have completed 90-day follow-up.

**3.6 Timing of Outcome Assessment**

- Complications: within 30 days post-surgery
- Mortality: 30- and 90-day
- Long-term oncologic endpoints: assessed during scheduled follow-up visits

**Section 4: Statistical Principles**

**4.1 Confidence Intervals and *p*-values**

- Effect estimates presented with 95% confidence intervals.
- Significance: *p* < 0.05, two-sided.
- No multiplicity adjustment; secondary analyses considered exploratory.

**4.2 Adherence and Protocol Deviations**

Patients receiving allocated intervention included in ITT. Major deviations recorded for PP analysis.

**4.3 Analysis Populations**

- **ITT:** all randomized patients
- **PP:** patients who adhered to assigned treatment without major deviations
- **As-treated:** patients analysed per actual surgery performed

**Section 5: Trial Population**

**5.1 Eligibility**

- Patients with clinically staged T_1-3_N_0-2_M_0_ tumours; good cardiopulmonary function;
- Patients with lower thoracic oesophageal tumours and oesophageal-gastric junction tumour;
- Patients without a previous history of cancer;
- Patients without a previous history of neck or chest surgery

**5.2 Withdrawal/Follow-up**

Withdrawal and lost-to-follow-up recorded; reasons documented.

**5.3 Baseline Characteristics**

Age, sex, BMI, tumour location, stage, neoadjuvant therapy, comorbidities summarized (Table 1).

**Section 6: Analysis**

**6.1 Outcome Definition**

- **Primary endpoint:** any complication (CD grade I–V) within 30 days
- **Secondary endpoints:** operative time, blood loss, number of lymph node dissections, three-day postoperative laboratory findings, length of hospital stay, operative-related 30-day and 90-day mortality after surgery; progression-free survival (PFS).

**6.2 Analysis Methods**

- **Primary endpoint:** chi-square/Fisher’s test; effect sizes as RR, OR, RD with 95% CI.
- **Complication severity distribution:** proportional odds logistic regression; if assumption violated, multinomial regression.
- **Continuous variables:** t-test or Mann–Whitney U.
- **Survival outcomes:** Kaplan–Meier, log-rank.

**6.3 Missing Data**

Primary analysis: ITT, complete-case. Sensitivity: worst-case imputation; multiple imputation if >5% missing.

**6.4 Harms**

SAEs coded and tabulated per CONSORT Harms extension; reported as counts and percentages by group.

**Supplementary Table S1. Clavien–Dindo classification of surgical complications**

| Grade | Definition |
| --- | --- |
| I | Any deviation from the normal postoperative course without the need for pharmacological treatment or surgical, endoscopic, or radiological interventions. Allowed therapies: antiemetics, antipyretics, analgesics, diuretics, electrolytes, physiotherapy. Bedside wound opening included. |
| II | Requiring pharmacological treatment with drugs other than those allowed for Grade I complications. Blood transfusions and total parenteral nutrition included. |
| IIIa | Requiring surgical, endoscopic, or radiological intervention not under general anaesthesia. |
| IIIb | Requiring surgical, endoscopic, or radiological intervention under general anaesthesia. |
| IVa | Life-threatening complication requiring ICU management with single organ dysfunction (including dialysis). |
| IVb | Life-threatening complication requiring ICU management with multiorgan dysfunction. |
| V | Death of the patient. |

**Supplementary Table S2. ECCG/ISDE definitions of esophagectomy-specific complication**

| Complication | Definition (ECCG/ISDE consensus) |
| --- | --- |
| Anastomotic leak | Full-thickness gastrointestinal defect at the esophagogastric anastomosis or conduit, demonstrated radiologically, endoscopically, or by drainage of digestive fluid/enteric content, or requiring intervention. |
| Pulmonary complications | Includes pneumonia (clinical signs, radiological infiltrate, plus positive sputum/bronchoalveolar culture or need for antibiotics), ARDS^b^, or respiratory failure requiring reintubation or prolonged ventilation (>48 h). |
| RLN^a^ palsy | Diagnosed by postoperative laryngoscopy showing vocal cord paralysis or immobility, or persistent hoarseness confirmed by ENT^c^ specialist. |
| Chyle leak | Milky drainage fluid with triglyceride concentration >110 mg/dL, or output consistent with enteral fat challenge, confirmed by laboratory testing or imaging. |
| Conduit necrosis | Ischemia or necrosis of the gastric conduit confirmed radiologically, endoscopically, or intraoperatively, requiring intervention. |
| Anastomotic stricture | Dysphagia requiring endoscopic dilatation, radiological evidence of narrowing, or inability to pass a standard endoscope. |
| Gastric outlet obstruction/delayed gastric emptying | Intolerance of oral diet with need for nasogastric drainage, radiological/endoscopic evidence of gastric stasis, or requirement of prokinetic therapy beyond 7 days. |
| Other surgical site infections | Superficial or deep wound infection defined by CDC^d^ criteria, requiring antibiotic treatment or drainage. |

a. RLN, recurrent laryngeal nerve; b. ARDS, acute respiratory distress syndrome; c. ENT, ear–nose–throat; d. CDC, Centres for Disease Control and Prevention.

**Supplementary Table S3. Summary of Serious Adverse Events (SAEs) According to CONSORT Harms Extension**

| **Serious Adverse Event** | **Definition / Criteria (Clavien-Dindo Grade ≥ IIIb)** | **McKeown MIE (n = 136)** | **Ivor Lewis MIE (n = 136)** | **P value** |
| --- | --- | --- | --- | --- |
| **Reoperation required** | Any unplanned return to the operating room under general anaesthesia | 5(3.7) | 3(2.2) | 0.47 |
| **ICU admission for life-threatening event** | Respiratory or circulatory failure requiring mechanical ventilation, vasoactive support, or reintubation | 16(11.8) | 23(16.9) | 0.23 |
| **Clavien-Dindo IVa/IVb complications** | Life-threatening organ dysfunction requiring intensive support | 3(2.2) | 0 | 0.08 |
| **Postoperative death (Grade V)** | Death within 90 days post-surgery | 4(2.9) | 1(0.7) | 0.49 |
| **Total serious adverse events** | Patients experiencing ≥ 1 SAE (non-exclusive) | 28(20.6) | 27(19.9) | 0.88 |

Data are presented as n (%) of patients. SAEs were defined as Clavien–Dindo grade IIIb–V events, including unexpected reoperation under general anaesthesia, ICU admission for life-threatening complications, or death, in accordance with the CONSORT Harms extension.

**Supplementary Table S4. Effect Sizes for Overall Postoperative Complications Between McKeown and Ivor Lewis Minimally Invasive Esophagectomy**

| **Outcome** | **McKeown (n=136)** | **Ivor Lewis (n=136)** | **Risk Difference (95% CI)** | **Risk Ratio (95% CI)** | **Odds Ratio (95% CI)** | **p value** |
| --- | --- | --- | --- | --- | --- | --- |
| **Any complication** | 81 (59.6) | 57 (41.9) | -0.176 (95% CI: -0.293 to -0.060) | 0.70 (95% CI: 0.55 to 0.90) | 0.49 (95% CI: 0.30 to 0.79) | 0.004 |
| **Anastomotic leak** | 23 (16.9) | 11 (8.1) | -0.088 (95% CI: -0.166 to -0.010) | 0.48 (95% CI: 0.24 to 0.94) | 0.43 (95% CI: 0.20 to 0.93) | 0.03 |
| **Anastomotic stenosis** | 31 (22.8) | 9 (6.6) | -0.162 (95% CI: -0.244 to -0.080) | 0.29 (95% CI: 0.14 to 0.59) | 0.24 (95% CI: 0.11 to 0.53) | <0.001 |
| **Pulmonary infection** | 28 (20.6) | 27 (19.9) | -0.007 (95% CI: -0.103 to 0.088) | 0.96 (95% CI: 0.60 to 1.55) | 0.96 (95% CI: 0.53 to 1.73) | 0.88 |
| **RLN palsy** | 5 (3.7) | 0 | -0.037 (95% CI: -0.069 to -0.004) | 0.091 (95% CI: 0.01 to 1.59) | 0.09 (95% CI: 0.01 to 1.58) | 0.06 |
| **Surgical site infection** | 8 (5.9) | 2 (1.5) | -0.044 (95% CI: -0.089 to 0.000) | 0.25 (95% CI: 0.05 to 1.16) | 0.24 (95% CI: 0.05 to 1.15) | 0.05 |
| **Blood transfusion** | 20 (14.7) | 26 (19.1) | 0.044 (95% CI: -0.045 to 0.133) | 1.30 (95% CI: 0.76 to 2.21) | 1.37 (95% CI: 0.72 to 2.60) | 0.33 |
| **ICU** | 16 (11.8) | 23 (16.9) | 0.051 (95% CI: -0.032 to 0.135) | 1.44 (95% CI: 0.80 to 2.60) | 1.53 (95% CI: 0.77 to 3.04) | 0.23 |
| **SAEs** | 28(20.6) | 27(19.9) | -0.007 (95% CI: -0.103 to 0.088) | 0.96 (95% CI: 0.60 to 1.55) | 0.96 (95% CI: 0.53 to 1.73) | 0.88 |

Risk ratio (RR), odds ratio (OR), and risk difference (RD) for the primary endpoint—any postoperative complication within 30 days after surgery—are shown with corresponding 95% confidence intervals. Effect sizes were estimated using binomial regression and logistic regression models in the intention-to-treat population.

**Supplementary Table S5. Distribution of Postoperative Complications by Clavien–Dindo Grade in the Two Study Groups**

| **Clavien-Dindo grade** | **McKeown (n=136)** | **Ivor Lewis (n=136)** | **Total (n=272)** |
| --- | --- | --- | --- |
| **0 (no complication)** | 55 (40.4) | 79 (58.1) | 134 (49.3) |
| **I** | 8 (5.9) | 5 (3.7) | 13(4.8) |
| **II** | 41 (30.1) | 31 (22.8) | 72(26.5) |
| **III** | 25 (18.4) | 20 (14.7) | 45(33.1) |
| **IV** | 3 (2.2) | 0 | 3(1.1) |
| **V (death)** | 4(2.9) | 1(0.7) | 5(1.8) |

The frequency and cumulative incidence of postoperative complications are presented by Clavien–Dindo grade (I–V) for the McKeown and Ivor Lewis groups. Between-group comparisons were assessed using the proportional odds model, adjusting for prespecified covariates (age, tumour location, clinical stage, and neoadjuvant therapy).

**Supplementary Table S6. Definition and Composition of Analysis Populations**

| **Population** | **Definition** | **Included (n)** | **Handling of crossovers/conversions** |
| --- | --- | --- | --- |
| **ITT** | All randomized patients | 272 (136+136) | Retained as randomized |
| **PP** | Excluded protocol deviations or conversions | 261 (130+131) | Excluded |
| **As-treated** | Analysed by actual procedure performed | 270 | Crossovers analysed per actual treatment |

Summary of the predefined analysis populations, including the intention-to-treat (ITT), per-protocol (PP), and as-treated sets. The table details inclusion criteria, the number of patients analysed in each population, and the handling of crossovers, conversions, and early terminations.

**Supplementary Table S7. Summary of Missing Data and Handling Procedures**

| **Variable** | **McKeown (n=136)** | **Ivor Lewis (n=136)** | **Total Missing, n (%)** | **Handling Method** |
| --- | --- | --- | --- | --- |
| Age | 0 | 0 | 0 (0%) | — |
| Body mass index (BMI) | 1 | 2 | 3 (1.1%) | Imputed |
| Neoadjuvant therapy status | 0 | 0 | 0 (0%) | — |
| Operative time | 0 | 1 | 1 (0.4%) | Complete-case |
| Postoperative complications (any) | 0 | 0 | 0 (0%) | — |
| Clavien–Dindo grade | 0 | 1 | 1 (0.4%) | Imputed |
| Length of hospital stay | 2 | 1 | 3 (1.1%) | Imputed |
| 30-day mortality | 0 | 0 | 0 (0%) | — |
| 90-day mortality | 0 | 0 | 0 (0%) | — |

The table summarizes the extent of missing data for key baseline and outcome variables in each study arm and the corresponding handling method. Missingness was uniformly low (<5% for all primary and secondary outcomes). Complete-case analysis was used for the main analyses. Multiple imputation by chained equations was performed as a sensitivity analysis, yielding consistent results with the complete-case findings.
